# Perceived Social Support and Medication Dose Interruption Among Pulmonary Tuberculosis Patients in Western India: A Cross-Sectional Study

**DOI:** 10.7759/cureus.74507

**Published:** 2024-11-26

**Authors:** Nirmal Ahuja, Ashley Kuzmik, Kristin Sznajder, Eugene Lengerich, N Benjamin Fredrick, Michael Chen, Wenke Hwang, Rajendra Patil, Bushra Shaikh

**Affiliations:** 1 School of Behavioral Sciences and Education, Penn State Harrisburg, Middletown, USA; 2 Penn State Ross and Carol Nese College of Nursing, Pennsylvania State University, State College, USA; 3 Public Health Sciences, Penn State College of Medicine, Hershey, USA; 4 Family and Community Medicine, Penn State College of Medicine, Hershey, USA; 5 Ophthalmology, Penn State College of Medicine, Hershey, USA; 6 Medicine, Mauli Hospital, Bhiwandi, IND; 7 Tuberculosis, Revised National Tuberculosis Control Program of India, Bhiwandi, IND

**Keywords:** adherence to medication, global health concern, interruption of treatment, medication noncompliance, perceived social support, social care, tb treatment adherence, the multidimensional scale of perceived social support, tuberculosis (tb)

## Abstract

Introduction

Despite efforts, tuberculosis (TB) remains a major public health problem in developing countries, and India alone accounts for most of the global TB cases. Although the treatment for TB is highly successful, a significant number of TB patients in India do not complete their assigned treatment. Social support has a key influence on medication adherence for chronic illnesses like diabetes, asthma, HIV, hypertension, cardiovascular diseases, and TB. However, limited research in India focuses on patients’ perceived social support and its association with medication dose interruption among pulmonary TB patients. This study aims to fill this gap and examine the association between perceived social support and medication dose interruption among pulmonary TB patients in Western India.

Methods

A cross-sectional study was conducted at three directly observed treatment, short-course (DOTS) centers in Bhiwandi City, Maharashtra state, in Western India. A total of 477 participants were recruited, and data on participants' medication dose interruptions in the last month, their clinical characteristics, and their perceived social support were collected. Descriptive and inferential statistics were applied.

Results

Perceived social support presented a significant association with TB medication dose interruption. Participants who reported low social support were significantly 2.6 times more likely to miss their doses in the last month (adjusted odds ratio (aOR) = 2.6 (95% confidence interval (CI): 1.556-4.403), P < 0.001). Participants who reported low family support were significantly 4.1 times more likely to miss their doses when compared with the participants who reported high family support (aOR = 4.1 (95% CI: 2.482-7.070), P < 0.001). Among the participants who missed their doses one to five times in the last month, those who reported low social support were significantly 2.5 times more likely to miss their doses, when compared with the participants who reported high social support (aOR = 2.5 (95% CI: 1.318-4.810), P = 0.005). Likewise, among the group of participants who missed their doses six or more times in the last month, those who reported low social support were significantly 3.1 times more likely to miss their doses, when compared with the participants who reported high social support (aOR = 3.1 (95% CI: 1.452-6.744), P = 0.004).

Conclusion

The study results underscore the importance of integrating patient-centered strategies into healthcare systems, ensuring that they not only offer social protection but also recognize social support as a critical component of TB treatment. This study could potentially inform various healthcare policymakers in formulating such patient-centric and focused intervention strategies by involving social networks and family dynamics.

## Introduction

In 2023, despite ongoing efforts, tuberculosis (TB) was globally the second leading cause of death from a single infectious agent after coronavirus disease [1]. Many factors such as poverty, inadequate access to health, stigma associated with TB, HIV co-infection, social determinant factors, and drug resistance make TB a persistent problem globally [1]. It continues to remain a major public health problem in developing countries, and among them, India shares the majority of the TB burden of the world, with 28% of the 10.6 million TB cases [1]. Worldwide, the treatment for TB is highly successful; however, due to the long duration of treatment, less than 30% of TB patients enrolled in India complete their assigned treatment [2]. Moreover, TB treatment typically involves a protracted course of antibiotics, and any interruptions or inconsistent adherence to TB medication can lead to treatment failure, drug resistance, and the continued spread of the disease [3]. Hence, many researchers have raised the concern about non-adherence and its potential implications.

More often, researchers have attempted to measure medication adherence mainly using medication dose interruption, which refers to any time throughout the entire treatment period that a patient missed a prescribed dose of TB treatment, ranging from missing even one dose in two weeks [4] to missing doses for at least two weeks, but less than two consecutive months [5]. However, understanding the factors that influence medication adherence in TB patients is much more pivotal to the global efforts to combat this ancient disease. Some of the major contributing factors to non-adherence to TB treatment in India have been the length of the TB treatment, high pill burden, and severe side effects of TB medications [6]. Furthermore, being adherent to the long course of TB treatment can be complex and a dynamic phenomenon due to the involvement of a wide range of demographic, social, and ecological factors affecting treatment adherence [7].

Realizing that the journey toward recovery from this disease extends beyond the confines of clinical settings, In the past 20 years, the connection between social support and health outcomes has garnered significant research interest in the fields of social and behavioral sciences [8]. Social support is a broad term, encompassing a variety of constructs, including support perceptions and receipt of supportive behaviors [9]. It also refers to a patient’s perceived and received support from his/her relationships within social network ties, such as familial relations, friends, neighbors, colleagues, fellow patients, health providers, or even social media connections [9]. Among these constructs, only perceived support has been regarded as consistently being linked to health [10]. Although studies in India recognize the value of social support and the need for developing focused social support interventions and social protection programs [7,11-13], few studies have examined perceived social support within social ties, such as family, friends, and others, and its influence on medication adherence.

This study potentially fills the identified gap and aims to examine and quantify the relationship between perceived social support and medication adherence among pulmonary TB patients in Bhiwandi City, India, with a focus on understanding how support from family, friends, and significant others influences the interruption of the medication doses.

## Materials and methods

Ethical considerations

This study was approved by the Institutional Review Board of Pennsylvania State College of Medicine (STUDY00010507). Written permission was also obtained from the city TB officer of the Revised National Tuberculosis Control Program (RNTCP) of Bhiwandi City. All patients provided written informed consent prior to data collection. All the surveys took place in a separate room to maintain the privacy and confidentiality of the conversation, and no one other than the surveyor and the participant was allowed to be present in the room. Clear and concise instructions were provided about the survey in the consent form and also during the survey process. The participants were also reassured during the survey that their answers would be kept confidential, especially for sensitive topics, to reduce social pressure and biases. 

Study design and settings

This cross-sectional study was conducted at three directly observed treatment, short-course (DOTS) centers in Bhiwandi City of Maharashtra state, in Western India. The three DOTS centers were located in Shanti Nagar, Gayatri Nagar, and Nayi Basti slum areas in Bhiwandi City. These DOTS centers conduct primary TB prevention activities, including screening and outpatient treatment administration services to TB patients in their respective catchment areas, and were purposefully selected due to a high influx of patients and recommended by the RNTCP officer of Bhiwandi City. As the authors represent different locations and institutions, we worked together by holding regular virtual meetings and keeping the lines of communication open through emails and messaging.

Sample size and power

Considering that less than 50% of the TB patients in India enrolled for their treatment completed the assigned treatment [2,14], we assumed the population proportion (p = 0.50), the Z-value corresponding to a 95% confidence interval was 1.96, power was set at 80%, and the margin of error (E) was set to 0.05. The formula applied for the sample size estimation was n= Z^2^. p. (1−p)​/E^2^. Based on this estimation, the required sample size for the study was 350, but our final sample included 477 participants. 

Inclusion and exclusion criteria

The inclusion criteria were as follows: 1) they must be age 18 years and above, 2) able to speak and understand Hindi language, 3) have been diagnosed with pulmonary TB more than a month ago, and 4) able to provide written and informed consent. The exclusion criteria were as follows: 1) they were registered at any other DOTS centers in the area except the three selected DOTS centers, 2) started their TB treatment less than one month, and 3) have severe debilitating conditions (e.g., neurological disorders such as Alzheimer's disease) that could limit the ability of the patient to understand the questionnaire and participate in the study.

Data collection procedure

Data were collected in two phases from September 2018 to October 2019 at the three selected DOTS centers. A total of 586 respondents were approached to participate in the study. Out of them, 47 were ineligible and 62 participants refused to participate. There was a total of 477 completed surveys with a response rate of 81%. The survey instrument was designed to identify the association between perceived social support and medication dose interruption and its frequency and was also translated into Hindi language by the principal investigator who is also a native Hindi speaker. The survey was interviewer-administered and was self-reported. Only those participants who visited the three DOTS centers at the point of data collection were recruited for the study. The participants were first approached by the local TB officer or a local health worker in the DOT center and were asked if they would like to participate in the survey. Once they agreed, they were asked to provide written informed consent. All the surveys took place in a separate room to maintain the privacy and confidentiality of the conversation, and no one other than the surveyor and the participant was allowed to be present in the room. Clear and concise instructions were provided about the survey in the consent form and also during the survey process. Since the questionnaire was translated and adapted in Hindi language, pilot testing of the survey was completed on 20 participants to identify potential biases in the wording, structure, or response options. Eventually, this feedback was used to refine the survey linguistically with no major changes. These 20 participants were not included in the final study sample.

Measures

Medication Dose Interruption and Its Frequency

Medication dose interruption and its frequency during the last month were measured using two questions in the survey instrument. The question on medication dose interruption was categorized in closed responses of Yes/No. The frequency of missing doses or interruptions was determined by looking at the interruption patterns among TB patients and was categorized into three groups: zero missed doses, missed one to five doses, and missed six or more doses in the last month [15].

Demographic Information

The sociodemographic information included age, gender, marital status (married or with a partner and single or without any partner), migrant status (a migrant was defined as a person who has migrated to Bhiwandi City (except Thane and Mumbai) for work), and socioeconomic class. The socioeconomic class was identified using the Kuppuswammy Scale [16]. This scale is a validated instrument that uses a consumer price index and categorizes the participant’s socioeconomic class into three categories (upper, middle, and lower).

Clinical Characteristics

A few clinical characteristics, which are related to the medication dose interruption, were also included. This includes duration of TB treatment, number of pills consumed per day, experience of side effects, and presence of any likely major depressive disorder. Depression was assessed using a validated patient health questionnaire - a two-survey instrument [17].

Social Support

Perceived social support among TB patients was assessed by using a 12-item Multidimensional Scale of Perceived Social Support (MSPSS). This validated survey instrument had the Cronbach's alpha coefficient between 0.85 and 0.91 [18]. MSPSS is a seven-point Likert-type scale consisting of 12 items scored between 1 “absolutely no” and 7 “absolutely yes.” The scale has three subscales of four items each to determine support from family, friends, and significant others. The lowest and highest scores of the subscales are 4 and 28, respectively. The lowest and highest total scores obtained by the sum of subscale scores are 12 and 84, respectively. The average total score is calculated, and any score in the range of 1-5 was considered as low perceived social support, whereas the scores from 5.1-7 were considered as high perceived social support.

Statistical analysis

The data were analyzed using IBM SPSS Statistics for Windows, version 26.0 (released 2018, IBM Corp., Armonk, NY). The following analytical strategies examined the association between perceived social support and medication dose interruption. First, descriptive statistics were used to present the sociodemographic characteristics. Second, the binomial logistic regression model was applied to identify the association between social support and medication dose interruption. Variables like sociodemographic characteristics (age, gender, marital status, migrant status, and socioeconomic class), clinical factors (duration of treatment, number of pills consumed per day, and presence of side effects), and depression were controlled in the model. Lastly, the binomial logistic regression was also applied to find associations between perceived social support and frequency of medication dose interruption in the last month. In both regression methods, the Wald test was used to determine potential associations at a significance level of p ≤ 0.05.

## Results

Descriptive results

Demographic Characteristics

The majority of the 477 participants were female (58%), married or living with a partner (70%), and migrants (70%). More than two-thirds (69%) were in the age group of 18-35 years, and based on the Kuppuswammy Scale [16], 76% of the participants belonged to the lower socioeconomic class, followed by 18% in the middle class and 6% in the upper class (Table 1).

**Table 1:** Sample characteristics of the study participants (N = 477)

Variables	Tuberculosis medication dose interruption		
	Total n (%)	Yes n (%)	No. n (%)
Total	477 (100)	109 (23)	368 (77)
Sociodemographic characteristics			
Gender			
Male	199 (42)	44 (9)	155 (32)
Female	278 (58)	65 (14)	213 (45)
Age in years			
18-35	330 (69)	70 (15)	260 (55)
36-49	77 (16)	27 (6)	50 (10)
≥50	70 (15)	12 (2)	58 (12)
Marital status			
Single	141 (30)	41 (9)	100 (21)
Married	336 (70)	68 (14)	268 (56)
Migrant status			
Migrant	267 (56)	61 (13)	206 (43)
Non-migrant	210 (44)	48 (10)	162 (34)
Socioeconomic Class			
Lower	361 (76)	86 (18)	275 (57)
Middle	86 (18)	16 (3)	70 (15)
Upper	30 (6)	7 (2)	23 (5)
Clinical characteristics			
Duration of the tuberculosis treatment			
Up to one year	295 (62)	61 (13)	234 (49)
More than one year	140 (29)	37 (8)	103 (22)
Number of pills consumed per day			
1-4	344 (72)	77 (16)	267 (56)
≥5	133 (28)	32 (7)	101 (21)
Have experienced side effects of TB medications			
Yes	325 (68)	80 (17)	245 (51)
No	146 (31)	28 (6)	118 (25)
Frequency of missed doses in the last month			
None	368 (77)	0 (0)	368 (77)
One to five times	66 (14)	66 (14)	0 (0)
Six or more times	43 (9)	43 (9)	0 (0)
Major depressive disorder likely			
Yes	197 (41)	52 (11)	145 (30)
No	280 (59)	57 (12)	223 (47)
Perceived social support			
Total social support			
Low	268 (56)	78 (16)	190 (40)
High	209 (44)	31 (7)	178 (37)
Family support			
Low	131 (27)	53 (11)	78 (16)
High	346 (73)	56 (12)	290 (61)
Friend support			
Low	344 (72)	86 (18)	258 (54)
High	133 (28)	23 (5)	110 (23)
Significant other support			
Low	121 (25)	36 (8)	85 (18)
High	356 (75)	73 (15)	283 (59)

Clinical Characteristics and Depression

More than half of the participants (62%) reported a treatment duration of up to one year and 29% reported a treatment duration of more than one year. More than two-thirds (72%) of the study participants shared that they consume about one to four pills per day for their TB treatment and 68% of the participants experienced some form of side effects from their TB medication doses (Table 1).

Among 109 (23%) participants who reported medication dose interruption in the last month, 61 participants (62%) were on treatment duration for up to one year, 77 participants (71%) reported a TB pill burden of one to four pills per day, 80 participants (74%) reported that they had experienced some form of side effects from their TB medications, and 52 participants (48%) were more likely to have a presence of a major depressive disorder (Table 1).

Medication Dose Interruption

Medication dose interruption in the last month was reported by 109 participants (23%). Among them, 61% of the participants missed their doses one to five times in the last month and 39% missed their doses six or more times in the last month (Table 1)

Perceived Social Support

Overall, low social support was reported among 56% of the participants. Further categorization of social support revealed that 28% reported low family support, 72% reported low friend support, and 25% reported low significant other support (Table 1).

Among the 109 participants who missed their doses, 78 participants (72%) had low social support, 53 participants (49%) reported low family support, 86 participants (79%) reported low friend support, and 36 participants (33%) reported low significant other support (Table 1).

Association between perceived social support and medication dose interruption

The participants who reported low social support were significantly 2.6 times more likely to miss their doses when compared to the participants with high social support (aOR = 2.6 (95% CI: 1.556-4.403), P < 0.001). Similarly, participants who reported low family support were significantly 4.1 times more likely to miss their doses when compared with the participants with high family support (aOR =4.1 (95% CI: 2.482-7.070), P < 0.001). Friend support and significant other support revealed no significant associations with medication dose interruption (Table 2).

**Table 2:** Association of perceived social support and medication dose interruption among pulmonary tuberculosis patients’ sample (N = 477) OR: odds ratio; CI: confidence interval; in adjusted analysis variables – sociodemographic characteristics, clinical characteristics, and presence of major depressive disorder were adjusted in determining this association; *p ≤ 0.05 statistically significant.

Variables	Tuberculosis medication dose interruption			
	Unadjusted		Adjusted	
	OR (95% CI)	p-value	OR (95% CI)	p-value
Total Social Support				
Low	2.3 (1.483-3.748)	<0.001*	2.6 (1.556-4.403)	<0.001*
High	Referent	-	Referent	-
Family Support				
Low	3.5 (2.241-5.525)	<0.001*	4.1 (2.482-7.070)	<0.001*
High	Referent	-	Referent	-
Friends Support				
Low	1.5 (0.956-2.658)	0.074	1.5 (0.878-2.752)	0.130
High	Referent	-	Referent	-
Significant Other Support				
Low	1.6 (1.029-2.619)	0.037*	1.6 (0.970-2.760)	0.065
High	Referent	-	Referent	-

Association of perceived social support with frequency of medication dose interruption

Among the participants who missed their doses one to five times in the last month, those who reported low social support were significantly 2.5 times more likely to miss their doses, when compared with the participants who reported high social support (aOR = 2.5 (95% CI: 1.318-4.810), P = 0.005). Likewise, among the group of participants who missed their doses six or more times in the last month, those who reported low social support were significantly 3.1 times more likely to miss their doses, when compared with the participants who reported high social support (aOR = 3.1 (95% CI: 1.452-6.744), P = 0.004) (Table 3).

**Table 3:** Association of perceived social support and frequency of medication doses interruption in the last month among pulmonary tuberculosis patients’ sample (N=477). OR: odds ratio; CI: confidence interval; variables – sociodemographic characteristics, clinical characteristics, and presence of major depressive disorder were adjusted in determining this association; *p ≤ 0.05 statistically significant. Referent group (dependent variable): participants who have zero missed doses.

Perceived social support	Frequency of tuberculosis medication dose interruption in the last month			
	Missed doses one to five times		Missed doses six or more times	
	OR (95% CI)	p-value	OR (95% CI)	p-value
Total social support				
Low	2.5 (1.318-4.810)	0.005^*^	3.1 (1.452-6.744)	0.004^*^
High	Referent	-	Referent	-
Family support				
Low	6.6 (3.432-13.010)	0.000^*^	2.5 (1.243-5.263)	0.011^*^
High	Referent	-	Referent	-
Friend support				
Low	1.0 (0.543-1.962)	0.923	5.2 (1.541-17.689)	0.008^*^
High	Referent	-	Referent	-
Significant other support				
Low	2.3 (1.258-4.484)	0.008^*^	1.1 (0.531-2.420)	0.746
High	Referent	-	Referent	-

Furthermore, among the participants who missed their doses one to five times in the last month, those with low family support were significantly 6.6 times more likely to miss their doses, when compared with the participants who reported high family support (aOR = 6.6 (95% CI: 3.432-13.010), P < 0.001). Likewise, among the group of participants who missed their doses six or more times in the last month, participants with low family support were significantly 2.5 times more likely to miss their doses when compared with the participants who reported high family support (aOR = 2.5 (95% CI: 1.243-5.263), P = 0.01) (Table 3).

Moreover, friend support was significantly associated only among the group of participants who missed their doses six or more times in the last month. Participants who reported low friend support were significantly 5.2 times more likely to miss their doses six or more times in the last month, when compared with the participants who reported high friend support (aOR = 5.2 (95% CI: 1.541-17.689), P = 0.01). Lastly, the significant other support was observed to be significantly associated only among the group of participants who missed their doses one to five times in the last month. Participants who reported low significant other support were significantly 2.3 times more likely to miss their doses one to five times in the last month, when compared with the participants who reported high significant other support (aOR = 2.3 (95% CI: 1.258-4.484), P = 0.008) (Table 3).

## Discussion

To the best of our knowledge, this is the first study in India that examined and quantified the association between perceived social support and medication dose interruption among pulmonary TB patients. The results highlight that people who have low social support are 2.6 times more likely to miss their doses when compared with patients who have high social support. This significant association between social support and medication dose interruption reinforces the findings from the globally existing literature on the role of social support in medication adherence for chronic illnesses like diabetes, asthma, HIV, hypertension, cardiovascular diseases, and TB [8,9,12,19-25]. The study results further assert that the presence of a strong support system can provide TB patients with the encouragement, motivation, and resources necessary to adhere to their treatment regimens, thereby reducing the risk of medication dose interruptions and bolstering their chances of full recovery [11,12]. Moreover, the already known medical, social, psychological, and emotional influence of the overall social support system on TB patients makes such a study even more critical [12].

The study results also highlight the importance of family, friends, and significant other’s support and quantify their association with missed doses and their frequency. TB treatment is often described by the patients as rough, agonizing, and even devastating [7]. Hence, chronic diseases like TB demand for a need to have a social support system in the form of family members or friends to supervise the patient’s medication schedule and concerns and at times provide the required emotional support [11,12,23,26]. Our study echoed similar views, and the results signify the important role that a family, a group of friends, or a significant other could play in medication adherence behavior among TB patients. Moreover, understanding this social support system could also help us understand patient-centric elements that are crucial in addressing their beliefs, attitudes, subjective norms, cultural context, and emotional health challenges [7,12].

In addition, to assert the role of social support, numerous physiological and psychological mechanisms have been proposed in the literature that help us explain how social support affects medication adherence [27]. This includes the stress-buffering effect that enables people to adopt tailored sick-role behavior and take proactive steps toward adherence [25]. The existing social networks among patients could provide the desired support via a series of relationships and interconnectedness that could influence their behavior and also potentially influence treatment adherence [28]. Contrarily, unhealthy social networks might also affect health negatively by adding stress that impairs the attitudes and behaviors required for adherence and potentially may lead to non-adherent behavior [29]. Furthermore, social network ties may even discourage or influence others from using certain medications, thereby reducing adherence [28]. Future studies on exploring this interconnectedness of social networks could serve to be useful.

Besides discussing the direct role of social support, it is also equally important to consider the social and cultural context of the society that could potentially influence the social support system of the individual and simultaneously affect the medication adherence behavior [19]. For example, in India, social and cultural contexts influence the gender-related differential experience of non-adherent behavior among TB patients [7]. Women experience isolation and discrimination in their homes, face rejection from their husbands and in-laws, and at times are even excluded from the "marriage market" altogether [12,19]. This gender disparity and patriarchal mindset embedded in the cultural context of society not only undermines the social support system for female TB patients but can also impose even stricter social constraints as they navigate through their treatment hurdles. Moreover, in such cases, the family support may also differ with regard to the marital status of a woman. Few studies in India have indicated that married women did not experience the same degree of family support as compared with unmarried women [12]. Hence, even family support varies depending upon our relationship and gender-related statuses. Thus, future research on understanding this cultural and gender-based differentiation of social support and its influence on medication adherence could be beneficial.

Furthermore, there are also different opinions about the categorization of social support in previous studies. Researchers have also categorized perceived social support into five different types of constructs: emotional support, practical support, behavioral support, instrumental support, and appraisal support [28]. Hence, with TB medication adherence in India, diverse constructs of social support may generate a different result and some interrelationships. For example, a systematic review identified that a greater degree of practical support was most consistently associated with greater adherence to medication, whereas evidence for structural or emotional support was less compelling [24]. Therefore, future research examining these different constructs of social support could prove to be beneficial for developing some focused patient-centric intervention strategies.

Finally, while social support is emphasized in the study as a crucial patient-centered approach to enhance treatment adherence, it cannot serve as the sole approach. Globally, early detection and treatment, limiting exposure, and increased funding and research on new drugs are crucial. This covers both fundamental and applied TB research, as well as the development of novel, shorter treatments and innovative new drug delivery options that could improve patient compliance [6]. In addition, it is equally important to approach this issue from a socioeconomic standpoint and expand on existing social protection, job security, and cash transfer schemes. These policy initiatives have been shown to be strongly linked to the control of TB [30]. Alongside convergence with multi-sectoral efforts to address poverty, undernutrition, unsafe housing, and indoor pollution, India also needs a pro-poor model of patient-centered care that includes nutritional, psycho-social, financial, and universal health coverage and social protection [12,22,30]

Although the study's novel contribution in examining perceived social support specifically in the context of TB treatment adherence in India, positions this as a significant contribution to global health research. However, there are several limitations that should also be acknowledged. First, the study used a cross-sectional design, limiting the ability to infer causality between the variables studied and it only identifies the correlation between perceived social support and the outcome variable of medication dose interruption. Second, the study relied on self-reported data, which may be subject to bias or inaccuracies mainly due to cultural variances in social support interpretation. Third, because the study concentrated on the patients who came to the three DOTS centers from the slum areas of Bhiwandi City, there is a chance that the monolingual Marathi-speaking local population was overlooked. Future studies should focus on exploring diverse groups including the local population. Fourth, social support experiences may differ as per the culture and other sociodemographic factors, and hence, caution should be exercised when extrapolating these findings to broader or more diverse populations and regions. Fifth, even though we have defined the interruption and frequency of missed doses, there is a large gap between adherent and non-adherent. Therefore, there must be a good number of participants who would have been missed by this categorization. Sixth, only patients who received treatment at the DOTS center were included in our study; patients who did not receive or complete the full course of treatment at the center or who had poor treatment outcomes but were unable to visit the center might have different viewpoints. Seventh, based on the MSPSS scale, this study has categorized perceived social support into family, friends, and significant other support. However, the family, friends, and significant other support were assessed only using four questions (MSPSS scale) in each group. Thus, this study might not be beneficial in estimating the effect size for research on these specific social support components (family or friends), and further research on an in-depth understanding of family and friends support could be beneficial. Lastly, in the future, more studies with longitudinal or mixed-method designs, inclusive sampling, or culturally adapted measurement tools for different linguistic groups could be much more beneficial in identifying targeted and culturally relevant interventions to address the issue of medication adherence among TB patients in India. 

## Conclusions

Acknowledging its generalizability limits, our findings identify a significant correlation between perceived social support and its constructs (family, friends, and significant other’s support) and medication dose interruption among pulmonary TB patients in Western India. The results underscore the importance of integrating patient-centric strategies into health systems that prioritize social support as a fundamental component of TB care. By leveraging social networks and family dynamics, healthcare policymakers can design targeted interventions and strategies to enhance medication adherence, ultimately improving health outcomes for TB patients in India. Such strategies could potentially include community engagement programs, peer support initiatives, and family involvement in care processes, addressing the unmet challenges of medication adherence and fostering a more supportive environment for TB patients. Finally, while the findings suggest that social support may play a role in adherence, further research across diverse settings and with longitudinal designs is needed to establish causality and broader applicability.
